# FALLS AND NUTRITIONAL RISK AMONG RURAL STATE RESIDENTS

**DOI:** 10.1093/geroni/igz038.1756

**Published:** 2019-11-08

**Authors:** Mariana Wingood, Nancy M Gell, Emily Tarleton

**Affiliations:** University of Vermont, Burlington, Vermont, United States

## Abstract

Vermont continues to have one of the nation’s highest fall rates and its rurality may be a contributing factor. The purpose of our study was to compare fall history and nutritional risk (a fall risk factor also associated with rurality) in participants from rural and metropolitan areas. We collected data at statewide community-based fall risk screenings. During the events, nutritional data was collected using the DETERMINE Your Nutritional Health Screening Tool Questionnaire. We used descriptive statistics (chi2) to examine the relationship between fall history, nutritional risk, and rurality. From 123 subjects, 67% were classified as rural residents. There was no relationship between fall history and nutritional risk (p=0.6). Compared to rural residents, a significantly higher percentage of those living in metropolitan areas reported falls (54% versus 35% p=0.05). However, metropolitan residents were not at higher nutritional risk (49% versus 54%, p=0.61). National nutritional risk rates are lacking, but food insecurity is associated with nutritional risk. Our overall reported high nutritional risk (20%) is higher than the prevalence of food insecurity, both nationally (11%) and in Vermont (9%). In conclusion, we did not identify a relationship between fall history and nutritional risk. We did find a higher percentage of metropolitan residents reporting falls. Furthermore, we identified that DETERMINE is a feasible nutritional screening tool to use at fall risk screenings. It can be used to identify community-dwelling older adults at nutritional risk, but it may not have the sensitivity to identify an association between nutritional risk and falls.

